# Association Between Alzheimer’s Disease and Periodontal Inflammatory Parameters: A Systematic Review

**DOI:** 10.4317/jced.62519

**Published:** 2025-03-01

**Authors:** Adriana Melo, Javier Flores-Fraile, Roberto Lo Giudice, Enrico Marchetti, José Nart, Alice Rose Greethurst, Francisco Real-Voltas, Francesco Tarallo, Cosimo Galletti

**Affiliations:** 1 School of Dentistry, Department of Integrated Dentistry, International University of Catalonia, Sant Cugat del Vallès, Barcelona, Spain; 2 Department of Surgery. University of Salamanca. Campus Miguel de Unamuno 37007, Salamanca. Spain; 3 Department of Human Pathology of adults and developmental age, Messina University, 98100 Messina, Italy; 4 Department of Life, Health and Environmental Sciences, University of l’Aquila, Piazzale Salvatore Tommasi 1, 67100 Coppito, Italy; 5 Department of Periodontology, International University of Catalonia, Sant Cugat del Vallès, Barcelona, Spain; 6 Associate Professor. Kore University of Enna, Faculty of Medicine and Surgery, 94100, Enna, Italy

## Abstract

**Background:**

The purpose of this systematic review was to analyze the current evidence of the potential correlation between periodontitis inflammatory parameters and Alzheimer’s Disease (AD).

**Material and Methods:**

A systematic review was conducted in accordance with the PRISMA guidelines. Electronic literature searches in PubMed, Medline, EBSCO, Scopus (ELSEVIER), Cochrane Library (Wiley) and Grey Library were conducted to analyze relevant references. Eligibility was based on inclusion criteria which included cross-sectional studies published after 2012.The rationale for selecting this temporal framework was grounded in the availability of studies from this period that aligned with the objectives and parameters of the review Authors independently selected the studies and extracted the data. Quality assessment was conducted under the Newcastle-Ottawa scale. The outcome variables were objectives, demographics, risks factors, dental statement, Pathogens, and conclusions.

**Results:**

The technique used was the comparison, pooling and study of different case studies (considered, or not, significant and/or representative). Out of 564 potentially eligible articles, 5 cross-sectional articles were included based on specific inclusion criteria such as being published after 2012, alignment with the study objectives, and focusing on Alzheimer’s disease and periodontal inflammatory parameters. All five studies highlight a higher prevalence of AD in women that increases in age. While four studies supported connection between AD and periodontal inflammatory parameters, one study found no plausible association. The quality assessment displayed a mean score of 10.8 (Range: 0 to 13), being the domain “selection” the highest ranked and the “comparability” the lowest.

**Conclusions:**

Despite some conflicting studies, most suggest a positive correlation between PD and AD, highlighting the necessity for further clinical and longitudinal research. Also, patients with AD exhibit poorer oral hygiene, which contributes to PD, emphasizing the need for comprehensive dental care. Factors such as genetics, lifestyle and age play a significant role in this association.

** Key words:**Alzheimer’s disease, Cytokines, Gingipains, Periodontitis, Porphyromonas gingivalis.

## Introduction

Throughout life, humans face different diseases that can change their lifestyle or even go unnoticed. Among the most common diseases in the sixth decade of life, causing dependence and disability, are neurodegenerative diseases (1-7). Recognized as some of the most limiting irreversible neurological conditions worldwide, it is estimated that around 55 million individuals suffer from some form of dementia, categorized depending on its etiology (4,6,8-10).

To name a few there is vascular dementia, Lewy Body dementia, and one of the most frequent types, found in 60-80% of cases: Alzheimer´s disease (AD) (1,2,9,11).

AD and related dementias (RD) have distinct clinical differences, where the deterioration of language and memory occurs earlier in AD than in RD, making it a progressive neurodegenerative disease (2,7). Apart from genetic risks factors, there are other modifiable, acquired factors, including physical inactivity, smoking, education level, dietary habits, hypertension, traumatic brain injuries, type II diabetes, among others. However, to date, the specific causal mechanisms have not been fully established, leading to its classification as a multifactorial disease, with a higher prevalence in women than men (1,5,10,11).

When analyzing the molecular changes, a distinctive feature of AD is the accumulation of β-amyloid plaques in the brain. These plaques consist of fragments of proteins called amyloid peptides that aggregate in the cerebral

neocortex, specifically Aβ-42. Similarly, abnormal deposits of neurofibrillary tangles (NFTs*) occur within brain cells. These tangles are mainly composed of a protein called Tau, which forms abnormal structures, interfering with signal transmission between neurons, causing synaptic dysfunction and neuronal death 84 or partial destruction. This eventually results in the progression of AD (1,5-7,9-12).

Similarly, an increase in the low-level inflammatory response is observed in the affected brain, driven by the prolonged release of proinflammatory cytokines by glial cells such as microglia. These cells are activated in response to β-amyloid plaques and release inflammatory chemicals, playing a prominent role in the development of cognitive impairment (2,7,9,13-15).

Although the underlying mechanisms of dementia have not been fully elucidated, evidence increasingly suggests that when the inflammatory stimulus is not resolved in a timely manner, it leads to excessive cytokine production. This creates a state where these constantly activated proinflammatory cytokines become excessively sensitive to new immunological triggers and respond in an exaggerated manner (12). Elevated levels of proinflammatory agents (cytokines), such as interleukin IL-1β, Tumor Necrosis Factor Alpha (TNF-α), and C-reactive protein, have been observed in dementia patients, with the distinctive feature that these levels worsen with age (2,5,7-10,12).

The deterioration of cognitive skills and the ability to perform daily life activities expose people with dementia to complications in their oral health routine, leading poor oral hygiene, dental caries, oral pathologies, tooth loss, lack of use of dental prostheses, and periodontitis (1,7-9,12,14-16).

In the quest to identify factors that contribute to preventing the development of dementia, some authors highlight a potential connection between systemic inflammation caused by periodontitis and the exacerbation of the neurodegenerative environment (3-7,9,11-13,17-26). Studies have found associations between periodontal disease (PD) in cognitively healthy older adults and neuropathological changes related to AD, including elevated levels of beta-amyloid (Aβ) in the brain and increased inflammatory and amyloid markers in blood. This suggests that oral inflammatory processes could precede the onset of AD (7).

The oral cavity has the second-highest concentration of microorganisms after the intestine, harboring more than 700 species of microbiomes. The connection between the oral-intestinal-cerebral axis is considered both direct and indirect evidence of the association of oral microorganisms with immune mechanisms in the brain, particularly regarding periodontal pathogens (8-10).

It is important to know the etiology of periodontal disease to deduce the possible link not only with AD but also with other pathologies. The main pathogens include: *Aggregatibacter actinomycetemcomitans*, *Porphyromonas gingivalis*, *Tannerella forsythia*, *Prevotella intermedia*, *Treponema denticola*, *Fusobacterium nucleatum* (6,7,12). According to studies in rodents, *Porphyromonas gingivalis* depends on the support of other opportunistic microorganisms to generate dysbiosis (7). Initially, it manifests as inflammation of the gums with bleeding (gingivitis), which, if not treated, progresses to inflammation of the periodontium.

Regarding clinical and radiographic characteristics, the formation of periodontal pockets, loss of clinical attachment (CAL), and reduction in alveolar bone level are observed (6). This disorder is complex to diagnose as it depends on the degree of progression and the severity stage of the damage. Some determinants of periodontal disease result from an insufficient host response to bacterial pathogen attacks, linked to genetic and environmental susceptibility factors, tobacco use, diabetes, and alcohol consumption (18). Additionally, according to the World Health Organization (WHO), the incidence of PD is quite high, affecting around 50% of the world’s population, with a higher prevalence in the elderly and correlating with age (7,18).

Authors suggest that periodontitis is related to the incidence and progression of AD and that chronic oral infections could promote inflammation, contributing to confusion and dementia. Although the underlying mechanism has not yet been fully determined, research on post-mortem brain tissues from AD patients has demonstrated the presence of Lipopolysaccharides (LPS*) from various bacteria, including *Porphyromona Gingivalis*, *Treponema denticola* y Chlamydia pneumoniae, which, as mentioned earlier, are some components of the oral microbiome in PD (6,9,27).

Different studies in animal models have shown that harmful periodontal bacteria can access the brain in mice and possibly contribute to the development of AD. The possible pathways through which *P. gingivalis* and other microbiomes influence the brain include blood circulation, a weakened blood-brain barrier due to aging, inflammation, or persistent infections, olfactory and trigeminal nerves, and direct access through perivascular spaces. The precise identification of these pathways is crucial to gaining a more complete understanding of the relationship between these two diseases (6,10,12).

It is crucial to highlight that oral epithelial cells, when repeatedly exposed to bacterial toxins such as LPS and gingipain (a cysteine protease secreted by *P. gingivalis*), release pro-inflammatory cytokines, including TNF-α, prostaglandin E2 (PGE2), interleukins 1β (IL-1β), and IL-6. These cytokines initiate a cascade of molecular events that ultimately result in gingival cell death (1,7-9,12,13,27).

Similarly, researchers have shown that levels of C-reactive protein (CRP), an indicator of systemic inflammation, significantly increase in the serum of patients affected by periodontal disease (3,12,28,29).

The relationship between periodontitis and dementia is a topic of growing interest in medical research. While an association has been established between periodontitis and other conditions, such as cardiovascular diseases, the relationship between periodontitis and dementia has yielded inconsistent findings in the literature (10,13,14,25,30-32). The possibility that periodontitis is a modifiable risk factor in the development of dementia is currently under investigation, as the inflammation and bacteria associated with periodontitis could play a significant role in AD (28).

This study aims to review and discuss the current literature, with the goal of providing a deeper understanding of the potential correlation between inflammatory parameters of periodontitis and AD. Given the growing evidence of the systemic effects of periodontal disease on overall health, including its potential contributions to neurodegenerative diseases like Alzheimer’s, the study explores the role of periodontal disease, particularly caused by *Porphyromonas gingivalis*, in the progression of AD by contributing to neuroinflammation and amyloid pathology. Furthermore, the study seeks to highlight the clinical importance of this connection in both cognitive function and dental health, in order to identify potential preventive strategies and develop therapeutic approaches in dentistry.

## Material and Methods

1. Search strategy and Focused Question

The search strategy employed in this systematic review was developed in accordance with the Preferred Reporting Items for Systematic Reviews and Meta-Analyses (PRISMA) guidelines (35) and is registered in PROSPERO under the number CRD42024489237.The review is guided by the PICO (36) framework to address the research question:

-PICO Framework:

“In patients with Alzheimer’s disease and periodontitis (P), how do periodontal inflammatory parameters (I) compare to those of individuals with periodontitis but without Alzheimer’s disease (C) in terms of periodontal inflammatory parameters (O)?”.

2. Information sources and search

A comprehensive literature search was conducted to identify scientific articles aligning with the study’s objectives, all the studies considered in the compilation of this review, no more articles were found after July 2023.

Researchers conducted a focused literature search for cross-sectional studies using the following databases: PubMed, Medline, EBSCO, Scopus (ELSEVIER), Grey Library and Cochrane Library (Wiley). These databases were selected due to their broad coverage of medical and health-related literature, ensuring access to a wide array of pertinent cross-sectional studies. A thoroughly detailed collection of peer-reviewed literature is offered by PubMed and Medline, while citation analysis in Scopus makes it easier to find significant studies. By providing a variety of health resources, EBSCO improves our search, and the Grey Library enables us to find unpublished research that would not be accessible through conventional means. Although the Cochrane Library primarily focuses on systematic reviews, it also includes relevant cross-sectional studies that contribute to our understanding of the topic.

Articles published in the English language were electronically searched by four independent reviewers (A.M, C.G, A.R.G. and J.F.F) until July 2023, with no limitations concerning dates of coverage and publication status, across the Medline/PubMed, EBSCO, Wiley, Grey Library.

The following key words were applied for MEDLINE/PubMed combined by Boolean operators (AND, OR, and NOT) following the MeSH terms: (((((((Alzheimer’s Disease[MeSH Terms]) OR (Dementia[MeSH Terms]))OR (Neurodegenerative Disease[MeSHTerms])) AND (Periodontitis[MeSH Terms])) OR (Periodontal Disease[MeSH Terms])) OR (Periodontal health[MeSH Terms])) OR (Gingipain[MeSH Terms])) AND (porphyromonas gingivalis [MeSH Terms])

For searching the remaining electronic databases, the key terms used were as follows:

- Alzheimer Disease AND Periodontitis OR Periodontal health OR Periodontal disease OR Periodontal conditions

- Alzheimer Disease OR Dementia OR Neurodegenerative disease AND oral inflammation

- Alzheimer Disease OR Dementia OR Neurodegenerative disease AND Gingipain OR *Porphyromonas gingivalis* OR cytokines

Initially, title and abstract were evaluated for all articles identified through database exploration, duplicates and unrelated publications were systematically excluded from consideration. Four examiners (A.M, C.G, A.R.G and J.F.F) independently chose the studies following the inclusion criteria for eligibility.

Consensus was reached to resolve any disagreements.

Finally, Full-text examination was carried out for articles deemed appropriate for the current systematic review, adhering to the specified criteria in  Table 1.

4. Data collection and method of analysis.

The selected articles were thoroughly analyzed to extract relevant data, including author details, publication year, country of origin, study design, objectives, associations between the diseases, participant characteristics, and specific outcomes. Heterogeneity across studies was carefully assessed, as variability in study designs, populations, and outcome measures posed a significant challenge. To account for this variability, A random effects model was employed in our analysis. This model was chosen because it assumes that the true effect size may differ across studies, providing more generalized effect estimates.

By using a random effects model, researchers were able to address the observed heterogeneity and offer conclusions that account for the inherent variability, unlike a fixed effects model which assumes all studies estimate a common underlying effect.

In line with this, certain studies were excluded from the analysis due to non-homogeneous data across key variables such as study design, population demographics, and outcome measures. This heterogeneity prevented us from conducting a meta-analysis, as pooling such disparate data would have led to unreliable or misleading results. Non-homogeneous data introduces complexity and variability that complicates the interpretation of pooled outcomes, making it difficult to draw statistically valid conclusions. Therefore, examiners opted for a systematic review, which allows for a qualitative synthesis of the findings while accommodating the diverse characteristics of the included studies. This approach ensures a comprehensive interpretation of the evidence without distorting the overall conclusions due to variability.

5. Quality and risk of bias assessment.

The quality of studies included in this systematic review was scored by two evaluators (A.M and A.G) using the Newcastle-Ottawa scale (NOS) modified for cross-sectional studies, with a score ranging from 0 to 13 points. The scale consists of three key domains of risk of bias assessment: (i) Participant selection, (ii) group comparability and (iii) outcomes measures. Given the relatively small number of studies included (n=5), no formal sensitivity analysis was conducted, and no outliers were removed. To ensure a comprehensive synthesis of the available evidence, all studies were retained in the review.

## Results

**Figure 1:**
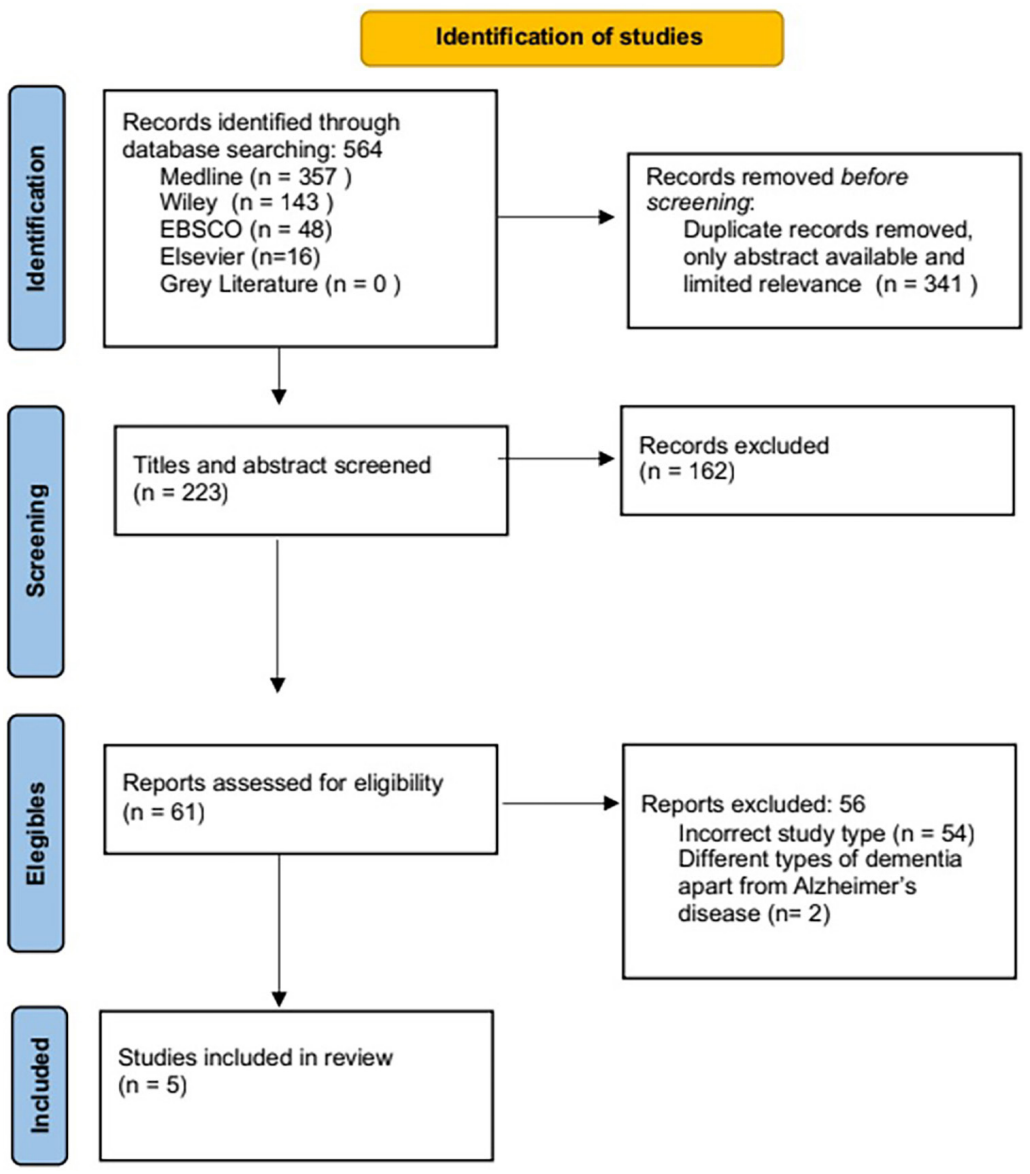
Search strategy flowchart.

To conduct this systematic review, the methodology for selecting research studies described in Figure 1 was followed. After conducting thorough research by using key words and MeSH terms, 564 published studies were identified:

Specifically, 317 from PubMed,143 from Wiley, 48 from EBSCO, 40 from Medline and 16 from Elsevier. Subsequently, 223 publications were obtained after eliminating articles with only abstracts available, irrelevant information and duplicate studies with the assistance of RefWorks tool. 61 articles were considered potentially eligible, after screening the title and the overall description of the abstract. 30 articles were selected based on comprehensive analysis of the full text matching the inclusion criteria, 25 articles were excluded at this stage, with exclusion reasons described in Fig. 1. Finally, 5 records were included for the development of this systematic review. A significant reason for exclusion was the presence of non-homogeneous data among the studies. Referring to the heterogeneity of the variables, populations, methodologies, and outcome measurements that can lead to inconsistencies in the results. For instance, studies that utilize different diagnostic criteria for diseases, varied age ranges, or dissimilar measurement tools for cognitive outcomes would not yield comparable results.

1. Study characteristics

All five studies included a minimum of 60 patients, aged 50 years and older. This decision is typically based on several scientific parameters presented on the selected studies, such as Age as a Risk Factor for Dementia and Periodontal Disease, prevalence of periodontal disease and study design considerations For instance, only five studies (n=5) are very limited and do not have good quantitative results to confirm the relationship between periodontal disease.

In total, 5.181 subjects were diagnosed with PD and with AD. Most of the studies were conducted on patients who only had AD; however, one study included participants who also suffered from diabetes, hypertension, cardiovascular and cerebrovascular disease. All the articles highlighted a higher prevalence of AD in women, which increases with age. Two studies reported that most of the intervention group lived alone or were institutionalized.

The mediators between PD and AD are described in the five articles, which indicate factors such as hypertension, diabetes, poor oral hygiene, tooth loss, malnutrition, respiratory disorders, hypertension, and cardiovascular diseases.

Four studies supported the relationship of AD and periodontal inflammatory parameters, while one study suggested that there is not a plausible association linking these two pathologies. Clinical periodontal parameters were assessed in each article, along with the pathogens identified in relation to both diseases.

A complete description of the selected cross-sectional studies concerning authors, year of publication, objectives, demographics, risks factors, dental statement, description of results and conclusions are shown in  Table 2. The Table contains research studies focused on periodontal health and its association with general cognitive decline. The studies specifically examine various populations, including individuals with Alzheimer’s Disease (AD), Mild Cognitive Impairment (MCI), and control groups, along with details about their corresponding age groups, dwelling situations, and risk factors. Additionally, the table provides a summary of the studies’ outcomes and conclusions.

In alignment with the PICO framework, the selected studies systematically address the population (P), intervention (I), comparison (C), and outcomes (O) relevant to the research question. The Table below ( Table 2) reorganizes and presents the study characteristics to explicitly reflect the PICO components, providing a clear and structured summary of the findings.

2. Assessment of the risk of BIAS All 5 studies were assessed by the modified and adapted NOS. The mean score was 10.8 (Range: 0 to 13), being the domain “selection” the highest ranked and the “comparability” the lowest, key validity aspects and quality are shown in  Table 3, Table 4.

## Discussion

AD and other dementias have been studied for several years. Since their emergence, various studies have been conducted to identify the causes of these diseases within the medical field. Throughout this research, different authors have associated certain proteins, which play important roles in the inflammatory and pathogenic processes found in periodontitis, with the progression of the brain changes that occur in dementia (1-4,8-12,16,18,20). This literature review has evaluated the possible relationship between periodontitis and various dementias, such as AD, based on an analysis of the most relevant existing studies to date.

It is important to highlight that dementia and AD are two distinct concepts; however, they are connected, as AD is the primary cause of dementia. The accumulation of deformed proteins, particularly β-amyloid and Tau, contributes to oxidative and inflammatory damage in the brains of older individuals, which characterizes AD. Dementia can be caused by different factors associated with various protein abnormalities, such as cerebral vascular alterations (1,5,6,11,12), leading to the loss of cognitive abilities.

As specified by Li, Mingui *et al*. and W. Micha *et al*., patients with dementia visit the dentist less frequently, with the vast majority presenting periodontal problems (1,3). Periodontitis is a chronic inflammatory disease that affects the supporting tissues of the teeth and is caused by bacteria such as *Porphyromonas gingivalis* and *Treponema denticola*, which accumulate in the oral cavity in the form of dental plaque. Various recent epidemiological studies have explored the possible association between periodontitis and dementia. While the results are not completely conclusive, most research suggests that periodontitis could be considered a risk factor for the development of AD (7).

Carballo and colleagues conducted a cross-sectional study in Spain with 90 patients, including 30 with AD, 30 with mild cognitive impairment (general cognitive decline), and 30 healthy controls. The results showed a higher prevalence of chronic periodontitis in Alzheimer’s and cognitive impairment groups compared to the control group. Additionally, levels of certain inflammatory markers, such as PCR, IL-6, and MMP-9, were significantly higher in serum in patients with EA and chronic periodontitis, suggesting a systemic inflammatory state (2).

Similarly, Ide *et al*. followed a cohort of 60 individuals with mild to moderate Alzheimer’s disease in the United Kingdom for six months. Those with periodontitis had a significantly higher rate of cognitive decline, along with elevated levels of the pro-inflammatory cytokines TNF-α and IL-10, although not of IgG against *P. gingivalis*. The authors suggest a possible role of systemic inflammation as a link between periodontitis and AD (19).

On the other hand, Li *et al*. conducted a cohort cross-sectional study on American patients over 65 years of age, comparing subjects with and without dementia and found a higher prevalence of severe periodontitis in the dementia group (9.1% vs. 5.2%). Additionally, dental care costs were 26% higher for patients with dementia. According to the authors, dementia is associated with worse outcomes in oral health (1). In other words, Li *et al*.’s research indicates an economic burden and utilization of dental care associated with AD and related dementias. This finding complements Carballo *et al*.’s study, which suggests an association between periodontitis and cognitive dysfunctions (2). Likewise, Wereszczyński *et al*. provide evidence of the relationship between periodontitis and specific memory processes, referring to past experiences at a specific time and place (3).

Case-control studies have also yielded results indicating that periodontitis represents a risk factor for cognitive decline and late-onset dementia, as Holmer *et al*. found that when comparing 105 Swedish patients with cognitive impairment to 33 healthy controls, severe periodontitis was significantly more prevalent in cases than in controls, even after adjusting for odds ratio (OR 4.5) (16).

In general terms, available epidemiological studies have found a relative risk of dementia greater than 1 in individuals with periodontitis compared to periodontally healthy individuals (1-4,7). A recent systematic review and meta-analysis by Nadim *et al*. included 12 studies with a total of 239,273 participants, with a pooled relative risk of dementia associated with periodontitis of 1.38 (95% CI 1.01-1.90) in cohort studies and 2.25 (95% CI 1.48-3.42) in case-control studies (11). Many of the reviewed articles agree that there is an encouraging pathophysiological correlation between periodontitis and AD.

Despite the results obtained by previous researchers, authors such as Syrjälä *et al*. did not find an association between periodontitis and specific types of dementia in their cross-sectional study, which evaluated 354 institutionalized elderly Finns. They found a higher prevalence of dental caries and poor oral hygiene in patients with AD. The authors argue that the cognitive changes typical of dementia could lead to the neglect of oral hygiene, confounding the possible causal relationship (20). Similarly, Jungbauer *et al*. agree in their narrative review, “Periodontal Microorganisms and Alzheimer Disease - A Causative Relationship?” that the existing epidemiological evidence is weak and contradictory, as some studies conducted on mice do not fully capture the complexity and variability of the disease as it occurs in humans with AD.

Therefore, they argue that a causal relationship between periodontitis and AD cannot be proven and requires more studies on the matter (5).

A cross-sectional investigation led by Marruganti and associates posits that periodontitis is partially influenced by its effect on hypertension among patients exhibiting cognitive decline. Nonetheless, they suggest that the bacterial migration observed in the brains of deceased AD patients warrants careful consideration (8,27). Together, these authors claim that the available epidemiological evidence seems to indicate a positive association between periodontitis and dementia; however, more clinical trials and longitudinal experimental studies are needed to prove the causality and directionality of this relationship (5,6,10,13,16,18,37).

Various biological mechanisms could explain the link between both diseases.

One possibility is that the systemic inflammation characteristic of periodontitis contributes to the pathogenesis of dementia (6). Periodontitis induces a local and systemic inflammatory response, with an increase in pro-inflammatory cytokines such as IL-1β, IL-6, and TNF-α (7). According to the inflammatory hypothesis of

AD, these cytokines could enter the brain, activating glial cells and promoting neurodegeneration through circulation in the bloodstream (6,9,19,27).

In fact, it has been observed that AD patients present elevated systemic levels of pro-inflammatory cytokines and C-reactive protein (3). Therefore, chronic systemic inflammation derived from severe periodontitis could exacerbate the neuroinflammation inherent to AD (6). Mao *et al*.’s systematic review also highlights the potential role of oral microbiomes in the development of Alzheimer’s disease (7). These findings align with those of Borsa *et al*. and Guo *et al*., who also suggest a relationship between periodontitis and AD (8,9).

Another possible mechanism involves the direct entry of periodontal bacteria into the bloodstream and subsequently to the brain. Species such as *Porphyromonas gingivalis*, *Tannerella forsythia*, and *Treponema denticola* have been isolated in brain tissue from AD patients (6,10,20,27,28,37). In particular, *P. gingivalis* can utilize infected leukocytes to spread and reach the central nervous system (8). Once in the brain, *P. gingivalis* can secrete gingipains, which are neurotoxic proteolytic enzymes (10). It has been confirmed that gingipains can dissociate neuronal proteins such as β-amyloid peptide and tau protein, promoting the formation of senile plaques/amyloid plaques and neurofibrillary tangles, which are distinctive features of Alzheimer’s disease (4,9).

Bacterial lipopolysaccharides also activate pro-inflammatory pathways inglia, contributing to neurodegeneration (3). Therefore, *P. gingivalis*, along with other oral pathogens such as *Tannerella forsythia*, *Treponema denticola*, and *Fusobacterium nucleatum*, could play a central role in the pathogenesis of Alzheimer’s disease (AD). These bacteria have been implicated in promoting chronic inflammation and neurodegenerative processes, suggesting that they may represent important targets for future therapeutic interventions aimed at slowing or preventing the progression of AD (4,10. Chuanjiang Zhao *et al*. support this biological mechanism in their research, indicating the positive outcome of Nisin (a polypeptide substance produced by a probiotic) in effectively counteracting these changes by altering the composition of the brain microbiome after periodontal infection, suggesting Nisin as a potential therapy in the prevention and treatment of AD (6). Shared genetic risk factors between periodontitis and Alzheimer’s have also been proposed (11), such as polymorphisms in genes related to innate immunity and inflammatory response (e.g., TLR4, NLRP3, TREM2). Patients with such polymorphisms would be more susceptible to both diseases (3).

Surprisingly, periodontitis not only seems to be related to neurodegenerative diseases, but it has also been postulated that periodontitis predisposes individuals to cardiovascular diseases, which in turn increases the risk of vascular dementia and various related dementias (12).

The strength of this study lies in its consideration of contemporary research from the past decade through comprehensive and methodologically sound search.The literature reviewed provides substantial evidence suggesting that periodontal disease may contribute to the development of Alzheimer’s disease, specifically through the inflammatory mechanisms associated with

*Porphyromonas gingivalis*, such as gingipains. This connection was partially supported by the results of four out of the five studies included in the review.

These studies identify significant correlations between periodontal disease and Alzheimer’s-related neuroinflammation and amyloid pathology, reinforcing the idea that periodontal health may influence the progression of Alzheimer’s disease.

However, the study by Syrjälä *et al*. (2012) did not support the observed association, as it did not find a strong link between periodontal disease and cognitive decline in Alzheimer’s patients. This inconsistency highlights the need for further research to explore the mechanisms underlying the relationship between these two conditions.  Table 5 summarizes the key findings of the studies

reviewed, providing an overview of the sample sizes and the observed relationships between periodontal disease and Alzheimer’s disease, while also emphasizing the variability in findings that necessitates further research to clarify the complex interactions between these conditions.

Additionally, our review contributes to an understudied area by fostering scientific curiosity and highlighting the clinical relevance of a potential, unexpected relationship between the two diseases, which may encourage further exploration and enhance understanding of their interaction.

These findings have important clinical implications for the healthcare team in clinical practice, as they not only motivate early periodontal diagnoses and raise awareness among patients from an early age about the systemic risks that poor oral hygiene habits may entail, leading to periodontitis, but also contribute from the field of dentistry to the prevention of this and other chronic diseases through our work. Although some available studies are observational in nature and therefore unable to establish causality between periodontitis and dementia (13), the vast majority do find an association between the two diseases. For this reason, some authors advocate for conducting clinical trials to test whether the prevention and treatment of periodontitis have a protective effect on cognitive decline and dementia (14). Within the limitations of the conducted research, it is important to note that there is an insufficient number of cross-sectional studies examining the relationship between Alzheimer’s disease and periodontitis, which limits the comprehensiveness of the evidence base. Additionally, the included studies exhibited a lack of homogeneity in terms of methodologies, populations, and outcomes, further complicating the possibility of conducting a robust statistical analysis. The low number of studies meeting inclusion criteria also restricts the generalizability of the findings, underscoring the need for more consistent and high-quality research in this area.

However, this research opens the door for future studies to focus on conducting RCTs and longitudinal studies examining the association between the severity of periodontitis and the severity of dementia. Considering each patient’s lifestyle, it may be possible to take a step further to ensure that periodontitis is recognized as one of the potential irrefuTable causes of degenerative diseases.

## Conclusions

The conclusions of this review indicate that Gingipains have been identified in Alzheimer’s brains serving as a key factor produced by *Porphyromonas gingivalis*, a bacteria associated with the periodontal inflammatory parameters; It is based on evidence from multiple studies that demonstrate its association with neuroinflammation and amyloid pathology in AD. However, investigators acknowledge the need for further statistical support to strengthen this conclusion.

Specifically, while several studies identified *P. gingivalis* in the brains of AD patients and observed its role in promoting neurodegeneration via gingipains, not all studies provided statistically significant results. To bolster the conclusion, researches cite studies such as Dominy *et al*. (2019) which offers robust data on the presence of *P. gingivalis* in AD brains and its pathogenic role, as well as recent systematic reviews and meta-analyses that highlight the correlation between periodontal pathogens and AD progression. Further research is needed to definitively establish statistical significance in this area.

Furthermore, the studies demonstrate that patients with Alzheimer’s and related dementias exhibit unfavorable oral hygiene compared to control groups, which ultimately leads to the development of periodontal disease. Therefore, comprehending this connection holds critical importance in formulating precise therapeutic interventions.

Several predisposing factors may impact this association; for instance, genetics play a significant role, such as unhealthy diets, smoking, sedentary lifestyles, and oxidative stress, also contributing to both conditions. Additionally, age is a common risk factor, as the prevalence of periodontitis and Alzheimer’s disease increases with advancing age.

Given these considerations, it is imperative to expand dental consultations beyond the exploration of the oral cavity as an isolated entity within the human body. Since periodontal disease is linked to various pathologies associated with systemic inflammatory processes, including Alzheimer, dentists should proactively advise and devise strategies for the care and maintenance of oral health. This includes reinforcing preventive treatments not only for patients with Alzheimer’s and their families/caregivers, but also for young adults at risk of developing either of these conditions, emphasizing the need for special attention and monitoring. While some studies have produced conflicting results regarding the association between periodontitis and Alzheimer disease, the majority suggest a positive correlation. Nevertheless, further clinical trial and longitudinal studies are needed to establish causality and determine the potential protective effect of periodontal treatment on cognitive decline and its progression.

Future expectations

In future research, it would be beneficial to compare the various stages of Alzheimer’s disease and the development of periodontitis across levels that were not addressed in the current systematic review. Additionally, conducting a longitudinal cohort study involving patients with Alzheimer’s disease, individuals at risk of Alzheimer’s disease without any pathology, and control subjects would help minimize confounding factors and facilitate long-term observation of disease progression and potential onset of pathologies.

Furthermore, the inclusion of articles presenting a variety of variables rendered statistical analysis unfeasible due to the lack of homogeneity in the collected data, thereby limiting the overall relevance of the systematic review.

## Figures and Tables

**Table 1:** Eligibility criteria.

INCLUSION CRITERIA	EXCLUSION CRITERIA
Articles published from 2012 and onwards	Studies in languages other than English
Full text available	Articles focused Just on the association of tooth loss and different dementias
Studies related with Alzheimer's disease and dementias.	Publications related with other type of dementias than Alzheimer´s
Alzheimer's and periodontitis related publications	Studies with objectives unrelated to investigating the association between Alzheimer's disease and periodontitis
Studies in patients above 50 years old	Author Comments
Patients above 50 years old with periodontitis	Protocols
Studies about treatments for the periodontal disease that could influence Alzheimer´s disease	Clinical trials
Cross-sectional studies	Case report studies

**Table 2:** Systematic evaluation of the five articles based on the review question.

Author / Year	Population (P)	Intervention (I)	Comparison (C)	Outcome (O)
Mingui L. et al. (2023)	Community dwelling older adults with AD and periodontitis.	Measurements made based on self-reports and classified as preventive/diagnosis events and definitive treatment events.	Community-dwelling older adults with periodontitis but without AD.	Patients with AD and periodontitis were more likely to have adverse dental care outcomes.
Marruganti et al. (2023)	Older adults with AD and periodontitis.	Full-mouth periodontal examination. Measurements of gingival margin position, PPD, CAL.	Older adults with periodontitis but without AD.	Moderate periodontitis was significantly associated with increased odds of low cognitive performance in all tests.
Wereszenzyńns ka et al. (2023)	Patients with AD and periodontitis.	Assessment of periodontal health and its relationship to specific memory processes/evaluation of cognitive markers.	Individuals with periodontitis but without AD.	Poor periodontal health is specifically linked to episodic memory issues, suggesting a potential correlation with AD.
Santosh et al. (2014)	Individuals with AD and periodontitis.	Evaluation of GI, PI, PD, CAL, and %BOP.	Individuals with periodontitis but without AD.	Periodontal health deteriorates with AD progression and is closely related to cognitive decline.
Syrjälä et al. (2012)	Individuals with AD and periodontitis.	Data collection through interviews and oral clinical examination.	Individuals with periodontitis but without AD.	No clear association between PD and AD; however, patients with AD are at increased risk of por oral health.

**Table 3:** Newcastle -Ottawa Quality assessment scale. 0 score: if the criterion is not met,1 score (*): partially met, 2 score (**): fully met.

	SELECTION			COMPARABILITY		OUTCOME	
STUDY	Sample description	Sample size	Surveillance tool	Confounding factor info	Clinic & demographic description	Independent Blind assessment	Discuss of limitations	Total	Major Limitations
Minghui et al. (2023)	**	*	*	*	*	**	**	10	-Inequitable sample between exposed and controls. -No information about dental statement of Alzheimer population studied -Survey taken without presence of dental professional to have accurate dental data
Marruganti et al. (2023)	**	**	**	**	*	**	**	13	-Additional information about clinical statements of the participants
Wereszczynski et al. 2023	*	*	**	*	*	*	**	9	-Participants were remunerated -There is not an exact number of patients with periodontitis -The description of the demographics and confounding factor information appears somewhat unclear and lacks specificity
Santosh et al. (2014)	**	**	**		**	**		10	-Limitations described are not related to the methodology or surveillance tools. -Not explicitly mention of confounding factors
Syrjälä et al. (2012)	**	*	**	**	**	*	**	12	-Inequitable sample between exposed and controls. -Study financially supported

**Table 4:** Study characteristics.Li, Minghi et al. Compared adults with Alzheimer’s Disease (AD) and those without Alzheimer’s and related dementias (ADRD). Findings indicated that individuals with AD had similar dental care but incurred higher economic costs. In contrast, individuals with dementia were less likely to visit the dentist, with 38% fewer visits overall.||Marrunganti,C et al. Comparison groups: Low cognitive performance prevalence and moderate to severe periodontitis showed significant associations but independent connection||Wereszczynski.M et al,Two comparison groups individuals with periodontitis and those without it, the presence of periodontal disease correlates with memory impairments that may indicate increased risk for cognitive decline with AD ||Martande,S et al. the comparison groups consisted of patients with Alzheimer’s and healthy individuals, Patients with AD had higher level of periodontal inflammation||SyrÄla,A et al. Comparison groups: AD patients, Vascular dementia and healthy patients, patients with AD are more likely to have severe periodontitis.

AUTHOR &YEAR	OBJECTIVES	POPULATION	AGE	COMPARISON GROUPS			DWELING		RISK FACTORS	DENTAL STATEMENT	AD-PERIODONTITIS MEDIATORS	OUTCOMES	CONCLUSIONS
Minghui et al. 2023	Determine the effects of Alzheimer´s disease (AD) and related dementias (RD) on dental care usage and economic burden in older adults	4.268 adults studied.	65-74yo	CONTROL	Female: 53.41%Male: 46.59%	94.48%	Living alone %	Institutionalised %	•Gingipains•Tooth loss•Suboptimal dental health behaviour (plaque accumulation•medication	•Caries 96%•periodontal disease53%•Xerostomia 29%•53% present moderate and severe periodontitis		→Alzheimer group:•Older adults • Higher prevalence in female•caregiver dependents•no medical insurance→comparison to adults without ADRD: AD: higher total outt-of-pocket dental care costs →RD group: 38% less likely to visit the dentist and 40% fewer visits	•Effective patient-centered strategies should be used to improve dental care outcomes in patients with ADRD.• Poor dental health observed in patients with ADRD might be due to greater functional dependence, increasing age,comorbidities and medication associated with ADRD
							41.69%	no reported					
			>85 yo	ALZHEIMER	Female: 68.61%Male: 31.39%	1.90%	46.09%	no reported					
			75-84 yo	RELATED DEMENTIAS	Female: 59.65%Male: 40.35%	3.63%	54.39%	no reported					
Marruganti et al.2023	To examine the epidemiological link between periodontitis and reduced cognitive performance in older adults, using a representative sample from the U.S. population.	2086 Adults	>60yo Mean age 68.6 YO	LOW COGNITIVE PERFORMANCE PREVALENCE AND PERIODONTITIS	Female: 1052Male:1034		no reported	no reported	•Hypertension linked to dementia.•Possible role of periodontitis as a risk factor for hypertension.•Periodontitis may elevate risk for other non-communicable diseases (NCDs) like diabetes and cardiovascular diseases.•Specifically, periodontitis correlates with hypertension and stroke risk.	NO/MILD PERIODONTITIS: N= 817 (1.40%) Global cognition score 10.5 (0.01)MODERATE PERIODONTITIS: N=989 (41.90%)Global cognition score 18.0 (0.01)SEVERE PERIODONTITIS: N= 280(8.60%)Global cognition score 24.9 (0.01)	•Hypertension•Diabetes•Cardiovascular or cerebrovascular disease •Biomarkers of systemic inflammation	Moderate and severe periodontitis showed significant associations with lower DSST* performance, with odds ratios of 1.66 and 2.97, respectively. Additionally, a one-millimetre increase in mean CAL was linked to lower performance in AFT*, DSST, and global cognition, with odds ratios of 1.44, 1.86, and 1.50, respectively.	The results of this study indicate an independent connection between periodontitis and diminished cognitive performance in older adults aged 60 years and above.
				Moderate periodontitis and low cognitive performance*p<.05,**p<.01,***p<.001	Global cognition score <-1.55Female: 2.22 (1.54-3.22)***Male:1.49 (1.03-2.16)*	overall:1.85 (1.42-2.49)***							
				Severe periodontitis and low cognitive performance	Global cognition score <-1.55Female: 3.70(1.80-7.59)**Male:2.15 (1.02-4.55)*								
				Mean PPD and low cognitive performance	Global cognition score <-1.55Base model:(B)1.83(1.11-3.01)						•B+Diabetes:1.82 (1.09-3.04)•B+ Hypertension: 1.70 (1.01-2.86)•B+Cardiovascular or cerebrovascular diseases: 1.83(1.11-3.02)		
				Mean CAL and low cognitive performance							B+Diabetes: 1.49 (1.14-1.98)B+ Hypertension: 1.45(1.09-1.92)B+Cardiovascular or cerebrovascular diseases: 1.50(1.14-1.98)		
Wereszczynski et al. 2023	1.Relationship between performance in comprehensive episodic memory tests and periodontitis. 2. Understanding the connection between oral health and cognitive abilities.	60 Adults	>65 years above	Gingivitis infection and subgingival calculus		CPITN1: 10% Of sextantsCPITN 2: 24%			•Malnutrition•Tooth Loss	Teeth with PPD >4mm (mean SD):CPITN1: .43(+/-sd).810CPITN 1.03 (+/- sd) 1.235	•Diabetes•Cardiovascular disease	The tangible signs and personal perception of poor oral health were associated with a lower tendency to engage in mind-wandering. This reinforces the Spontaneous Recovery Deficiency hypothesis.	Poor periodontal health seems to be specifically linked to episodic memory problems, while there is no solid evidence of a relationship with other mental abilities.
				Periodontitis satatus and mind wandering (ALZHEIMER: 5.52% older more advanced	Female:82%	CPITN 3: 46%CPITN 4: 20%261 sextants evaluated	100% living independently			Teeth with PPD >4mm (mean SD): CPITN 3: 2.02 (+/-sd)1.479CPITN 4:0.85(+/-sd)1.162261 sextants evaluated			
Santosh et al. 2014	To assess and contrast the periodontal health status of individuals with and without Alzheimer's disease (AD).	118 with at least 12 teeth to score	50 to 80 years	CONTROL:60	Female:34/60=56.7%Male:26/60=43.3%	Group 1:Normal individuals	no reported	no reported	Good:8/60 = 13%Fair:18/60=30%Poor:34/60=57%	Patiens with more that 12 teeth: 16.2 (+/- SD) 4.2Teeth with PPD >4mm (mean SD):(P<.05)Group 1: 2.39(+/- sd)0.5CAL: (P<.05)Group 1: 2.76(+/- sd) 0.55	Cardiovascular disease, diabetes mellitus and Parkinson disease malnutrition, poor hygiene,pressure ulcers, respiratory disturbancestooth loss	Individuals with Alzheimer's disease demonstrated heightened periodontal parameters, indicating a correlation between AD progression and deteriorating periodontal health. Significant differences were observed in key indicators, highlighting the potential interplay between Alzheimer's disease and periodontal status.	The periodontal health status of individuals with AD deteriorates with disease progression and is closely related to their cognitive function.
				ALZHEIMER : 58 individuals	Female:32/58=55.2%Male: 26/58=44.8%	Group 2,Mild: n=22 (13 female,9 male)Group 3, Moderate: n=18 (10 females,8 males)Group 4 Severe: n=18 (9 female,9male)			Good:2/58=3%Fair:16/58=27.5%Poor:40/58=69%	Patiens with more that 12 teeth:15.8(+/- sd) 3.6Teeth with PPD >4mm (mean SD) Group 2: 3.18 (+/- sd) 0.35Group 3: 3.99(+/-sd)0.32Group 4: 5.02(+/-sd)0.56CAL: Group 2: 3.58 (+/- sd) 0.37Group 3:4.52 (+/-sd)0.38Group 4: 5.58(+/-sd)0.58			
Syrjälä et al. 2012	To investigate the relationship between diagnosed dementia and oral health in individuals over 75 years of age, considering the specific type of dementia	354 intervention group for oral clinic examination	>75 and above	CONTROL278			53.20%	1.80%	36.6% poor oral hygine3.90% Smokers	n=154 participants Teeth with PPD >4mm (mean SD) 2.9(3.7) Teeth	As a result of poor oral health, cognitively impaired elderly people have Poor nutritional status and aspiration pneumonia	Dentate individuals among patients with:•Alzheimer's Disease 37% -Poor oral hygiene 78% -Poor denture hygiene 75%	•Patients with Alzheimer's and other dementias are more likely to have cavities, severe periodontitis, and poor oral hygiene compared to people without dementia. •The specific type of dementia does not significantly influence oral health.
				AlZHEIMER: 49	Female: 83.7%Male: 16.3%		40.80%	32.70%	77.8% poor oral hygine2%smoker	Teeth with PPD >4mm (mean SD) n=18 Participants2.8(3.3) Teeth			
				OTHER DEMENTIAS: 27			27.30%	54.40%	66.7% poor oral hygine				

**Table 5:** sssss

STUDY	Sample size	Findings	Support for conceptual Model
Minghui et al. (2023)	60	Found a higher prevalence of Alzheimer's disease (AD) in patients with periodontal disease (PD).	Strong support: Highlights the potential link between periodontal disease and AD.
Marruganti et al. (2023)	75	Reported significant associations between low cognitive performance and moderate to severe PD.	Moderate support: Suggests that cognitive decline may be influenced by periodontal health.
Wereszczyński et al. (2023)	60	Identified a correlation between periodontal disease and memory impairments, indicating increased AD risk.	Strong support: Reinforces the conceptual model by showing how periodontal disease may contribute to cognitive decline.
Santosh et al. (2014)	60	Found that AD patients exhibited higher levels of periodontal inflammation.	Moderate support: Demonstrates the inflammatory aspect of the model, but further exploration is needed.
Syrjälä et al. (2012)	89	No significant correlation between periodontal disease and AD progression, though higher PD prevalence in AD patients.	Limited support: Contradicts the model's expectation of a direct correlation, indicating a need for further exploration.

## Data Availability

The data presented in this study are available on request from the corresponding author.
